# Three-dimensional mapping and functional analysis of sympathetic innervation in aortic perivascular adipose tissue

**DOI:** 10.1093/lifemeta/loag015

**Published:** 2026-06-08

**Authors:** Zhao-Ning Wang, Yan-Jue Song, Liang Tan, Zhen-Yu Xu, Ting Meng, Dai-Chen Yao, Yang Liu, Shu-Wen Qian, Qi-Qun Tang, Yan Tang

**Affiliations:** Key Laboratory of Metabolism and Molecular Medicine, Ministry of Education, Department of Biochemistry and Molecular Biology of School of Basic Medical Sciences and Department of Endocrinology and Metabolism of Zhongshan Hospital, Fudan University, Shanghai 200032, China; Key Laboratory of Metabolism and Molecular Medicine, Ministry of Education, Department of Biochemistry and Molecular Biology of School of Basic Medical Sciences and Department of Endocrinology and Metabolism of Zhongshan Hospital, Fudan University, Shanghai 200032, China; Department of Neurology, Jiaxing Hospital of Traditional Chinese Medicine Affiliated to Zhejiang Chinese Medical University, Jiaxing, Zhejiang 314001, China; Key Laboratory of Metabolism and Molecular Medicine, Ministry of Education, Department of Biochemistry and Molecular Biology of School of Basic Medical Sciences and Department of Endocrinology and Metabolism of Zhongshan Hospital, Fudan University, Shanghai 200032, China; Key Laboratory of Metabolism and Molecular Medicine, Ministry of Education, Department of Biochemistry and Molecular Biology of School of Basic Medical Sciences and Department of Endocrinology and Metabolism of Zhongshan Hospital, Fudan University, Shanghai 200032, China; Key Laboratory of Metabolism and Molecular Medicine, Ministry of Education, Department of Biochemistry and Molecular Biology of School of Basic Medical Sciences and Department of Endocrinology and Metabolism of Zhongshan Hospital, Fudan University, Shanghai 200032, China; Key Laboratory of Metabolism and Molecular Medicine, Ministry of Education, Department of Biochemistry and Molecular Biology of School of Basic Medical Sciences and Department of Endocrinology and Metabolism of Zhongshan Hospital, Fudan University, Shanghai 200032, China; Key Laboratory of Metabolism and Molecular Medicine, Ministry of Education, Department of Biochemistry and Molecular Biology of School of Basic Medical Sciences and Department of Endocrinology and Metabolism of Zhongshan Hospital, Fudan University, Shanghai 200032, China; Key Laboratory of Metabolism and Molecular Medicine, Ministry of Education, Department of Biochemistry and Molecular Biology of School of Basic Medical Sciences and Department of Endocrinology and Metabolism of Zhongshan Hospital, Fudan University, Shanghai 200032, China; Key Laboratory of Metabolism and Molecular Medicine, Ministry of Education, Department of Biochemistry and Molecular Biology of School of Basic Medical Sciences and Department of Endocrinology and Metabolism of Zhongshan Hospital, Fudan University, Shanghai 200032, China

**Keywords:** perivascular adipose tissue, sympathetic nerves, norepinephrine, cold-induced hypertension, volume fluorescence imaging

## Abstract

Perivascular adipose tissue (PVAT) is a critical regulator of vascular homeostasis, and sympathetic nerves play a fundamental role in vascular function. However, the function of PVAT as an intermediary in neurovascular communication remains poorly understood. Due to the limitations of conventional two-dimensional (2D) imaging and the low tyrosine hydroxylase signal observed at room temperature, we reevaluated the sympathetic neuroanatomy of aortic PVAT (aPVAT) using volume fluorescence imaging under cold conditions. This approach enabled whole-mount three-dimensional (3D) visualization of the sympathetic network in murine aPVAT. Retrograde tracing was performed to identify neural origins. Cold-exposed mice were assessed for sympathetic activity, plasma norepinephrine levels, and blood pressure fluctuations. The function of aPVAT sympathetic nerves was further examined via local ablation with 6-hydroxydopamine. Our results revealed an undescribed, hierarchically organized sympathetic network, characterized by a primary nerve trunk along the aortic arch that branches into secondary fibers penetrating into the adipose tissue. This innervation exhibited a significant increase in density under cold exposure. Retrograde tracing confirmed the left stellate ganglion as the predominant source, which was shared by major thoracic organs such as the heart and lung. Importantly, local ablation of sympathetic nerves within aPVAT abolished the cold-induced hypertensive response, while ablation of sympathetic nerves within inguinal white adipose tissue had no such effect on hypertension. These findings established sympathetic nerves within aPVAT as a critical source of perivascular innervation and identified this localized neuro-adipovascular circuit as a potential therapeutic target for neurogenic hypertension.

## Introduction

Perivascular adipose tissue (PVAT), the adipose depot enveloping blood vessels, is a pivotal regulator of vascular homeostasis. It functions not merely as a structural support but also as an active endocrine organ that regulates cardiovascular metabolism by secreting bioactive mediators [1, 2]. Under physiological conditions, PVAT releases protective bioactive mediators such as nitric oxide (NO) and adiponectin to regulate vascular tone and suppress inflammation [3, 4]. Conversely, impaired PVAT under pathological conditions secretes harmful mediators, exacerbating vascular dysfunction [5, 6]. Our previous work also demonstrated that bone morphogenetic protein 4 (BMP4)-mediated PVAT browning can prevent vascular constriction [7]. Furthermore, increasing evidence indicates that metabolically activated PVAT, characte­rized by a brown-like phenotype with enhanced thermogenic activity, can preserve vascular homeostasis through anti-inflammatory action [8]. Beyond these well-established secretory functions, the identification of substantial norepinephrine (NE) stores within PVAT [9, 10] raises a critical question regarding its sympathetic innervation. Given its anatomical proximity to blood vessels, PVAT may be positioned to serve as a key interface for neuro-vascular crosstalk.

The activity of vascular tone is precisely regulated by the sympathetic nervous system (SNS) [11, 12]. It extensively innervates blood vessels and releases NE, which acts primarily on adrenergic receptors on vascular smooth muscle to induce vasoconstriction [13, 14]. This mechanism represents a fundamental pathway in the pathogenesis of hypertension and related cardiovascular diseases. Despite this, conventional paradigms of neurovascular regulation have predominantly focused on direct nerve-to-vessel interactions, largely overlooking the potential modulatory role of the adjacent PVAT [15]. This oversight is particularly significant given the intimate anatomical association of PVAT with the vasculature and its emerging role as a site for neuro-adipose crosstalk. PVAT contains substantial stores of NE and expresses catecholamine-metabolizing enzymes and catecholamine uptake transporters, indicating a local capacity for NE uptake, handling, and modulation [16–18]. These features, coupled with early histological observations of tyrosine hydroxylase (TH)-positive signals within PVAT, strongly suggest the presence of sympathetic innervation within PVAT [19]. However, this perspective is challenged by recent high-resolution imaging studies, which report that sympathetic innervation in PVAT is generally sparse and exhibits a predominantly vasocentric distribution, even in regions such as the aortic PVAT (aPVAT) where nerve fibers appear relatively more abundant [20]. Consequently, the precise anatomical distribution and functional significance of sympathetic nerves in PVAT remain contentious.

Sympathetic innervation is a well-established regulatory pathway in other adipose depots. In brown adipose tissue (BAT), sympathetic fibers originate from the stellate and thoracic chain ganglia (T1–T5) [21]. These fibers form dense arborizations that directly innervate brown adipocytes to drive uncoupling protein 1 (UCP1)-dependent thermogenesis in response to cold or leptin signaling [22]. Similarly, in white adipose tissue (WAT), sympathetic inputs from the celiac ganglion extensively branch to contact adipocytes, promoting lipolysis and cold-induced beiging [23]. Such neural mapping not only regulates local metabolic functions but also enables cross-talk through circulating factors, such as fatty acids and leptin [24, 25]. Thus, the sympathetic neuroanatomy and functional roles in these classic adipose depots are well-defined. Parallel to this metabolic regulation, the thoracic aorta and heart are governed by a well-defined autonomic circuit: sympathetic outflow to the aorta originates primarily from the stellate ganglion (SG), while sensory feedback is conveyed to the central nervous system (CNS) via the dorsal root ganglia (DRG) and nodose ganglia (NG) [26, 27]. PVAT resides precisely at the anatomical intersection of these two regulatory domains: adipose and the cardiovascular. However, its sympathetic innervation remains poorly defined. We hypothesize that a functional sympathetic network may exist within PVAT and participate in cardiovascular regulation, but it may have been overlooked in prior studies due to methodological constraints or limited examination under resting physiological conditions.

In this study, exploiting the volume fluorescence imaging technique, we revealed a hierarchical sympathetic innervation pattern within aPVAT, characterized by a primary sympathetic trunk along the aortic arch. We further traced its origin to the SG. To elucidate the functional significance of this discovery, we utilized cold exposure as a classic sympathetic stimulus. The results illustrated that cold exposure significantly activated aPVAT sympathetic activity concomitant with a rise in blood pressure (BP). Most importantly, through chemical ablation of aPVAT sympathetic nerves with 6-hydroxydopamine (6-OHDA), we demonstrated that this local sympathetic activation is essential for the development of cold-induced hypertension. These findings collectively defined aPVAT as a sympathetic effector tissue and established its local neural network as a critical modulator of vascular function. This provides more evidence for understanding neuro–PVAT–vascular communication.

## Results

### A hierarchically organized sympathetic network is visualized by the volume fluorescence imaging technique

**Figure 1:**
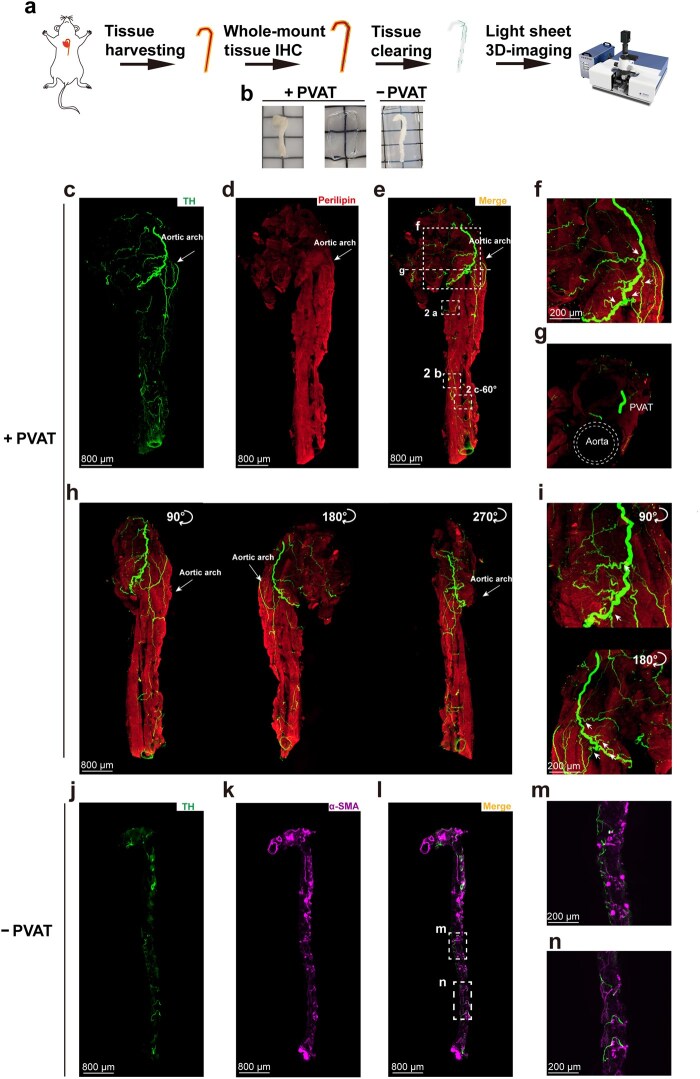
The hierarchically organized sympathetic network visualized by volume fluorescence imaging technique. (a) Flowchart of the whole-mount immunolabeling and volume fluorescence imaging of PVAT. (b) The aPVAT of the wild-type mice before (left) and after (middle) whole-mount immunolabeling and tissue optical clearing, and vessels without aPVAT of the wild-type mice (right). (c–e) Volume images of aPVAT immunolabeled for TH (488 nm; sympathetic nerves) and perilipin (594 nm; adipocytes). (f) Magnified images of sympathetic nerves surrounding the aortic arch, obtained from different angles. (g) Representative image of aPVAT in the horizontal plane. (h) Volume image of aPVAT co-immunolabeled for TH (488 nm; sympathetic nerves), perilipin (594 nm; adipocytes), and DAPI (405 nm; nuclei) at the 360° rotational views. (i) Local enlargement images of sympathetic nerves at the aortic arch from different angles. (j–n) Volume images of vessels without aPVAT immunolabeled for TH (488 nm; sympathetic nerves) and α-SMA (647 nm; vascular smooth muscle).

To investigate the sympathetic innervation of aPVAT, we employed a volumetric imaging approach based on the iDISCO+ protocol [28]. The tissue was processed through cyclic dehydration-rehydration for whole-mount immunolabeling, followed by organic solvent-based optical clearing (Fig. 1a). After clearing, aPVAT became nearly transparent, enabling volume fluorescence imaging on the light sheet microscope (Fig. 1b). Utilizing this approach, we sought to examine the sympathetic innervation in aPVAT by immuno-labeling TH, the marker for sympathetic nerves. Volume fluorescence imaging of anti-TH immuno-labeling demonstrated the presence of sympathetic nerves in aPVAT at single-fiber resolution (Fig. 1c; Supplementary Movie S1). Co-immunolabeling with the adipocyte-specific marker perilipin confirmed the sympathetic innervation within aPVAT (Fig. 1d, e, and h; Supplementary Fig. S1a). Furthermore, we identified a distinct organizational architecture characterized by a primary large-diameter sympathetic trunk coiling along the curvature of the aortic arch (Fig. 1e). This trunk was observed traversing along the aortic arch in immediate proximity to the aPVAT surface (Fig. 1f and i). Then this primary trunk subsequently branched into secondary small-diameter fibers, thereby establishing a pattern of “primary trunk to secondary branches” (Fig. 1f and i). Images of transverse plane confirmed that this trunk coursed in tight association with the adipose tissue (Fig. 1g). This anatomical location of this primary trunk was further verified by direct ste­reomicroscopic examination (Supplementary Fig. S1b). To further clarify the relationship between this sympathetic network and the vasculature, we imaged vessels after careful removal of the surrounding aPVAT (Fig. 1b). While TH-positive fibers were present on the α-smooth muscle actin (α-SMA)-positive adventitial surface (Fig. 1j–l), their density was markedly lower compared to the innervation observed within the intact aPVAT (Fig. 1m and n). This reduction indicated that a substantial proportion of the sympathetic innervation visualized in aPVAT was physically contiguous with aPVAT.

These findings extend previous reports of a vessel-centric innervation pattern in PVAT [20]. We provide a more comprehensive view of sympathetic innervation within aPVAT, revealing a hierarchical sympathetic network that had been previously undescribed. The hierarchical organization, particularly along the baroreceptor-rich aortic arch, which is subjected to significant hemodynamic forces [29, 30], may represent an anatomical substrate for sympathetic modulation of vascular function through aPVAT.

### Unique distribution characteristics of sympathetic innervations in aPVAT

**Figure 2:**
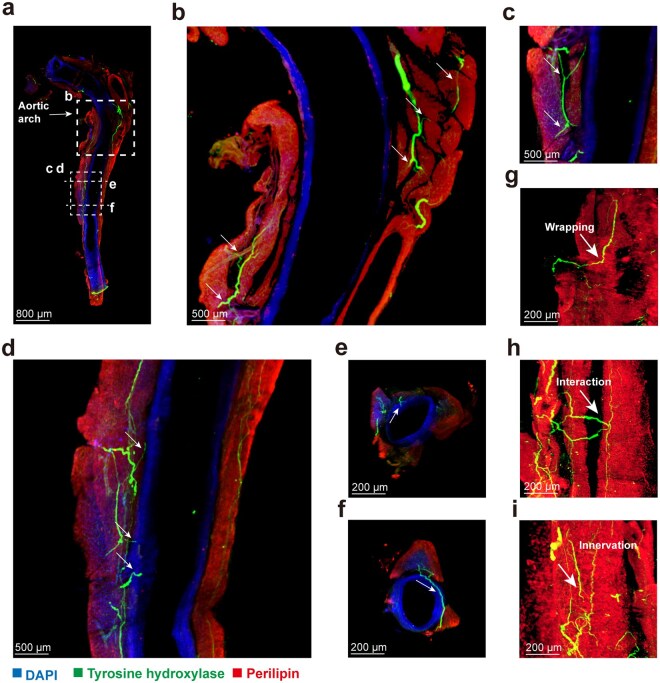
Unique distribution characteristics of sympathetic innervations in aPVAT. (a–c) Representative 3D projections of aPVAT in the sagittal planes which show sympathetic nerves (TH, 488 nm) forming direct neuro-adipose interfaces within adipocytes (perilipin, 594 nm). (d–f) Orthogonal planes demonstrating sympathetic fibers (arrows) traversing aPVAT: sagittal and horizontal planes. (g–i) Diverse distribution patterns of secondary branches under cold exposure: wrapped around aPVAT surfaces (g), connected transversely between adjacent aPVAT (h), or directly innervated adipocytes (i).

This volume fluorescence imaging technique enabled detailed mapping of sympathetic distribution within aPVAT. We observed that the secondary, small-diameter nerve fibers arising from the primary trunk also exhibited the traversing pattern. They traversed through the adipose lobules and branched into the adipose tissue (Fig. 2a–c). Horizontal plane imaging further confirmed the vessel-centric pattern (Fig. 2d), as a number of sympathetic nerves were observed traversing aPVAT to reach the blood vessel (Fig. 2d–f). Furthermore, these secondary fibers exhibited diverse distribution patterns. They either wrapped around aPVAT surfaces to form a superficial neural network (Fig. 2g), connected adjacent aPVAT to establish potential inter-aPVAT communication channels (Fig. 2h), or extended into aPVAT to directly innervate adipocytes (Fig. 2i). We further quantified the distribution patterns of sympathetic nerves. Results indicated that fibers exhibiting a direct innervating pattern were the most abundant, followed by those interconnecting with each other or wrapping around aPVAT surfaces (Supplementary Fig. S1c). This quantitative profile supports the presence of a complex innervation within aPVAT, which may underlie its specific function.

These anatomical patterns, including traversing, enveloping, and interconnecting configurations, indicate that aPVAT serves as more than a passive conduit for sympathetic nerves, but rather represents an extensively innervated effector tissue with complex neuro-adipose integration.

### SG origin of sympathetic nerves in aPVAT

**Figure 3:**
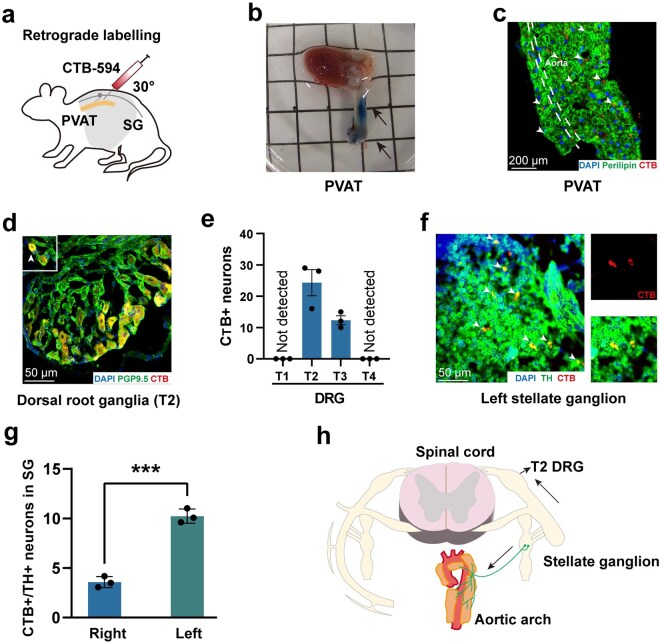
Stellate ganglion origin of sympathetic nerves in aPVAT. (a) Schematic diagram of the retrograde tracing experiment in which CTB is injected into aPVAT. (b) Trypan blue dye successfully injected into aPVAT at the low angle method. (c) Successful CTB deposition (594 nm) in aPVAT confirmed by co-labeling with adipocyte marker perilipin (488 nm). (d) Representative image of CTB-labeled (594 nm) T2 DRG section, co-labeled with PGP9.5 (488 nm) and DAPI (405 nm). (e) Quantification of CTB-positive neurons in thoracic DRGs (*n *= 3). (f) Representative image of a CTB-labeled (594 nm) left stellate ganglion section, co-labeled with TH (488 nm) and DAPI (405 nm). On the right are magnifications with the bottom-right panel displaying the overlay of the three channels. (g) Quantification of CTB-positive, TH-positive cells in the left and right SG (*n *= 3). (h) Proposed structural mechanisms of neural regulation in aPVAT. *P < 0.05; **P < 0.01; ***P < 0.001 by Student’s *t*-test or ANOVA test.

To determine the origin of neural populations innervating aPVAT, we performed retrograde tracing by locally injecting Alexa Fluor 594-conjugated cholera toxin subunit B (CTB) into aPVAT at a low angle of 30° to minimize diffusion. This noninvasive injection method allowed for neural circuit tracing under physiological conditions (Fig. 3a). The feasibility of this approach was confirmed in preliminary experiments using trypan blue, which showed localized distribution within the aPVAT (Fig. 3b). Successful delivery and uptake of CTB were verified by the presence of CTB-positive signals within targeted aPVAT (Fig. 3c). Subsequently, CTB-labeled sensory neurons were detected in thoracic DRG (Supplementary Fig. S2a–d), predominantly in T2 ganglia (Fig. 3d and e), confirming previously reported sensory innervation of PVAT [31]. On the other hand, CTB-labeled sympathetic neurons were observed in the SG (Fig. 3f; Supplementary Fig. S2e–g), consistent with the predominant sympathetic inputs [26, 27]. Although CTB-positive signals were present in bilateral SGs, these labeled neurons exhibited higher colocalization with TH-positive neurons in the left SG (Fig. 3g), indicating a left-dominant lateralization in the sympathetic origin. This provided a structural basis for its role in sympathetic modulation (Fig. 3h). In addition, we detected weak CTB-positive signals in the NG, suggesting that aPVAT also received sparse input from vagal nerve fibers (Supplementary Fig. S2h).

### Sympathetic nerves in aPVAT are activated under cold exposure

**Figure 4:**
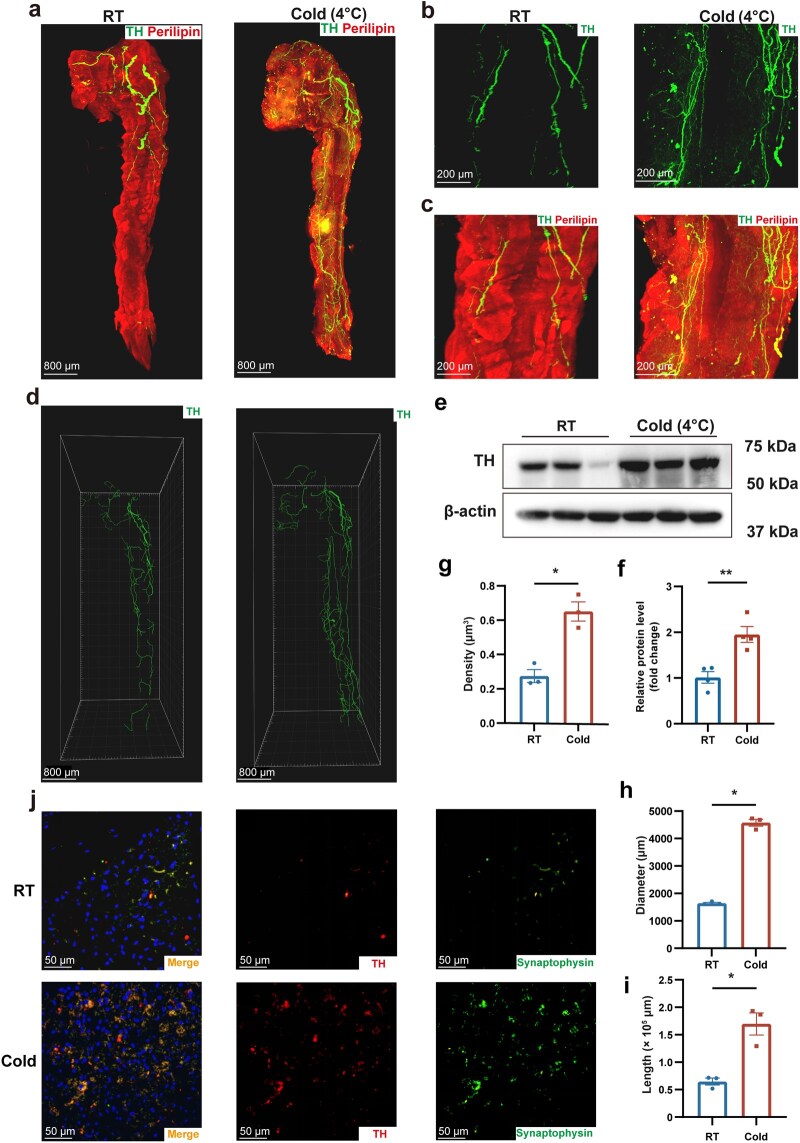
Sympathetic nerves in aPVAT are activated under cold exposure. (a–c) Representative 3D projections of aPVAT under room temperature (22°C–23°C) or cold exposure (4°C), immunolabeled by anti-TH and anti-perilipin. (d, g–i) 3D reconstruction of TH (488 nm) in each group. Quantification of density (g), diameter (h), and length (i) are shown (*n *= 3). (e and f) Western blot analysis and quantification of TH levels in aPVAT from mice maintained at room temperature (22°C–23°C) or exposed to cold (4°C) (*n *= 4). (j) Representative fluorescence images of TH (594 nm) and synaptophysin (488 nm) in aPVAT from mice maintained at room temperature (22°C–23°C) or exposed to cold (4°C). *P < 0.05; **P < 0.01; ***P < 0.001 by Student’s *t*-test or ANOVA test.

Based on the anatomical evidence of a sympathetic network within aPVAT and its origin from the SG, we hypothesized that cold exposure may activate aPVAT-localized sympathetic nerves. To test this, we subjected mice to 4°C for either 3 h or 72 h. As occurs during typical cold exposure, the expression of thermogenic genes in aPVAT increased, confirming the effectiveness of cold exposure (Supplementary Fig. S3e–g). Compared with mice maintained at room temperature (22°C–23°C), cold-exposed mice exhibited a time-dependent sympathetic activation, with more pronounced effects observed after 72 h. Therefore, we selected the 72-h time point for further investigation (Fig. 4a; Supplementary Fig. S3a). Detailed images clearly showed the increased density in sympathetic nerve fibers (Fig. 4b and c). Three-dimensional (3D) quantification confirmed the significant increase in sympathetic nerve density after cold exposure (Fig. 4d, g–i). These nerve fibers displayed activated morphological features, including increased density (Fig. 4g), thickened diameter (Fig. 4h), and elongated length (Fig. 4i), suggesting enhanced terminal branching and increased neurotransmitter release capacity.

At the molecular level, cold exposure elevated both the mRNA and protein levels of TH, the rate-limiting enzyme in NE synthesis in aPVAT (Fig. 4e and f; Supplementary Fig. S3b). Consistent with these findings, hematoxylin and eosin (H&E) staining showed increased density of TH-positive nerve fibers in aPVAT (Supplementary Fig. S3c). To further distinguish whether cold exposure affects the structure of nerve endings rather than simply inducing TH protein overexpression, we examined sympathetic innervation by immunolabeling synaptophysin. Synaptophysin is an integral membrane protein of presynaptic vesicles and has been widely used as a specific marker for synaptic vesicles and presynaptic terminals. Fluorescence images demonstrated that synaptophysin expression in aPVAT increased after cold exposure and showed significant colocalization with TH, confirming the dense distribution of sympathetic nerve terminals under cold conditions (Fig. 4j).

Collectively, these results demonstrate that cold exposure induces structural and molecular remodeling of sympathetic nerves within aPVAT. This remodeling is characterized by nerve fiber proliferation, morphological changes, and enhanced capa­city for localized NE synthesis, positioning aPVAT as a responsive sympathetic effector tissue during cold stress.

### Local sympathetic ablation within aPVAT abolishes cold-induced hypertensive effects

**Figure 5:**
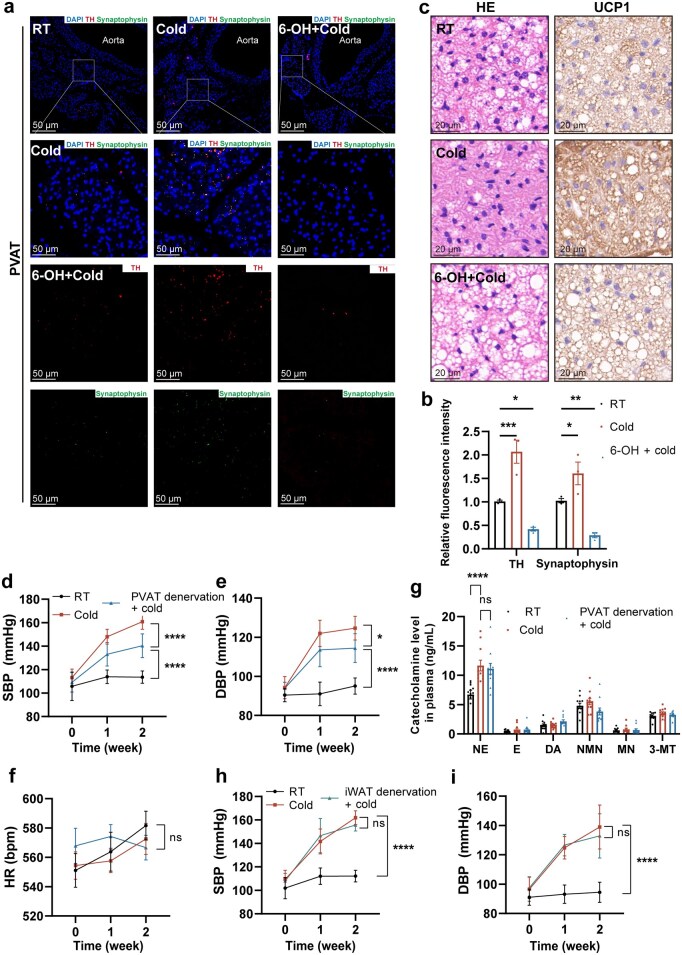
Local sympathetic ablation within aPVAT attenuates cold-induced hypertensive effects. (a and b) Representative fluorescence images of TH and synaptophysin (a), and quantification of fluorescence intensity (b). The aPVAT of wild-type mice were locally treated with saline or 6-OHDA under room temperature and cold exposure. (c) Representative images for H&E staining and UCP1. The aPVAT of wild-type mice were locally treated with saline or 6-OHDA under room temperature and cold exposure. (d) SBP of aPVAT denervation (*n *= 11). (e) DBP of aPVAT denervation (*n *= 11). (f) HR of aPVAT denervation (*n *= 11). (g) Catecholamine concentrations in the plasma from mice under room temperature or cold exposure (4°C) (*n *= 11). (h) SBP of iWAT denervation (*n *= 11). (i) DBP of iWAT denervation (*n *= 11). *P < 0.05; **P < 0.01; ***P < 0.001; ****P < 0.001 by Student’s *t*-test or ANOVA test.

Accumulating evidence has confirmed that sympathetic nerves in PVAT directly modulate vascular tone through NE release [19, 32], highlighting this pathway as a potential therapeutic target for hypertension. However, the aPVAT-specific contribution of the sympathetic network to cold-induced hypertension has yet to be clarified. To address this, we locally injected 6-OHDA into aPVAT to selectively ablate sympathetic innervation. Immunofluorescence (IF) analysis confirmed a marked reduction in TH and synaptophysin signals, indicating successful sympathetic denervation (Fig. 5a and b). Furthermore, in response to cold challenge, 6-OHDA-treated aPVAT lost the typical multilocular beige adipocyte morphology and exhibited reduced UCP1 expression compared to saline-treated and cold-exposed controls (Fig. 5c). Consistent with clinical reports on cold-associated cardiovascular risk [33, 34], intermittent cold exposure (4°C, 4 h/day for 2 weeks) induced a sustained increase in BP, while 6-OHDA treatment successfully abolished the cold-induced hypertensive response, as shown by a significantly attenuated rise in both systolic BP (SBP) and diastolic BP (DBP), with heart rate (HR) unchanged (Fig. 5d–f). We further measured plasma catecholamine levels. After cold exposure, the NE levels in plasma increased significantly (Fig. 5g), which strongly correlated with the rise in SBP (*R *= 0.7972, *P *< 0.05; Supplementary Fig. S4a). Notably, ablation of sympathetic nerves in aPVAT did not significantly affect plasma NE levels (Fig. 5g). Moreover, the correlation between plasma NE fluctuations and BP changes was no longer significant (*R *= −0.4387, *P *> 0.05; Supplementary Fig. S4b). This suggests that the BP-lowering effect of aPVAT sympathetic nerve ablation primarily acted on local mechanisms rather than by affecting circulating NE levels. Furthermore, to assess the specificity of this effect, we performed parallel sympathetic denervation in inguinal WAT (iWAT), a subcutaneous fat depot not associated with major vessels. In contrast to aPVAT ablation, sympathetic denervation of iWAT had no significant effect on the development of cold-induced hypertension (Fig. 5h and i; Supplementary Fig. S4c). This result confirms that the anti-hypertensive effect was specific to the sympathetic innervation of aPVAT.

Collectively, these data demonstrate that aPVAT-specific sympathetic activation is essential for the development of cold-induced hypertension. Traditionally, the aorta has been viewed primarily as an elastic conduit to maintain blood flow, while minute-to-minute BP regulation is mainly attributed to arterioles. However, recent studies positioned the aortic BP as a superior predictor of hypertension progression [35, 36]. Our finding that targeted modulation of aPVAT can influence the development of hypertension may provide new evidence for this perspective.

## Discussion

This study, using the volume fluorescence imaging technique, reveals a previously undescribed hierarchical sympathetic network within aPVAT. This architecture is characterized by a primary trunk along the aortic arch from which secondary fibers branch extensively. Critically, we further demonstrate that this localized neural innervation originates from the left SG. This sympathetic network can be activated by cold exposure and is essential for cold-induced hypertension.

### Reevaluation of sympathetic innervation in aPVAT

Our anatomical findings provided a new perspective on the debate regarding the sympathetic innervation in PVAT. Conventional two-dimensional (2D) imaging methods could only offer limited resolution of TH fragmentally [19, 37]. A recent high-resolution study reported limited innervation of PVAT, suggesting that nerves are primarily vessel-associated [20]. Although this network appeared relatively limited under physiological conditions, consistent with prior observations, we demonstrated that cold exposure triggered its significant activation, marked by a substantial increase in density and structural remodeling. Our whole-tissue 3D visualization did not contradict this observation but rather provided a more comprehensive perspective. We observed nerve fibers traversing the aPVAT to reach the vascular adventitia, substantiating a vessel-centric pattern. More importantly, we revealed a hierarchical sympathetic arborization within aPVAT which differed from the innervation of other classical adipose depots. In BAT and WAT, sympathetic nerves form a relatively homogeneous, diffuse network that densely contacts adipocytes [21, 23]. By contrast, the sympathetic network in aPVAT exhibits marked regional heterogeneity, with primary trunks entering at the aortic arch and branching. Secondary branches are organized not as a uniform network but as a defined hierarchical trunk-to-branch pattern, a configuration resembling a specialized conduction and distribution pathway rather than a dense terminal effector network. Furthermore, beyond the direct innervation pattern seen in WAT and BAT [21, 23], we observed a diverse spatial distribution in aPVAT: in addition to directly innervating adipocytes, sympathetic fibers also wrapped around aPVAT surfaces and connected adjacent aPVAT [21, 23], enabling more rapid and efficient signal transmission. These findings redefine aPVAT as an active neuro-adipovascular interface. The sympathetic network may leverage its perivascular position to be a critical amplifier in vascular regulation, distinct from β_3_-adrenoceptor-mediated thermogenesis in WAT or BAT [22, 24]. The thoracic aorta is precisely regulated by a classical autonomic neural circuit. In this circuit, afferent signals are conveyed to the CNS via sensory nerves and vagal afferents, with their cell bodies in the DRG and NG, which in turn can modulate sympathetic outflow [26]. The efferent sympathetic fibers originate from preganglionic neurons in the spinal cord and synapse in the paravertebral sympathetic chain. The inferior cervical ganglion often fuses with the first thoracic ganglion to form the SG in the thorax [27]. Our retrograde tracing revealed that the primary sympathetic input to aPVAT originated from the SG, with a pronounced left-sided predominance. This suggests an asymmetric sympathetic regulatory mechanism that may be critical for precise modulation of aortic arch hemo­dynamics. Additionally, we detected labeling in the NG, indicating vagal projections, and in the DRG, indicating sensory input. These findings imply that aPVAT is not merely an efferent target but may also sense local mechanical or chemical changes, positioning it as a potential integrator within broader autonomic and sensory reflex arcs.

### Functional complexity: the sympathetic innervation of aPVAT in cold-induced hypertension

An interesting finding of this study is the critical contribution of aPVAT-specific sympathetic drive to cold-induced hypertension. Acute cold exposure disrupts autonomic balance [38], causing sympathetic overactivation, which further leads to vasoconstriction and elevated arterial stiffness [39, 40]. This process impairs the sensitivity of baroreceptors such as those in the carotid sinus, weakening the inhibitory feedback to sympathetic centers and thereby establishing a self-sustaining vicious cycle [41, 42]. Our work identified sympathetic activity within aPVAT as a critical local node and potential therapeutic target within this cycle. Previous *in vitro* studies on mesenteric PVAT have suggested that under physiological conditions, sympathetic nerves within PVAT mediate an anti-contractile effect, likely through NE uptake and release of vasodilatory factors such as NO and adiponectin [19, 43, 44], while our *in vivo* findings show that ablating aPVAT sympathetic nerves abolishes cold-induced hypertension, indicating that under cold exposure, these nerves exhibit a contractile effect. To reconcile these observations, we propose a context-dependent model of aPVAT sympathetic regulation. During intense and sustained sympathetic stimulation, such as cold exposure, profound activation of the aPVAT sympathetic network leads to substantial local NE release, which may saturate PVAT uptake mechanisms. The resultant NE spillover then directly activates adrenergic receptors on vascular smooth muscle, triggering vigorous vasoconstriction, increasing vascular stiffness, and amplifying sympathetic output. In this high-output state, aPVAT thus shifts from acting as a protective buffer to a contractile amplifier. Although cold exposure also induces aPVAT browning [45], a process generally considered vasoprotective, the direct pro-contractile drive overrides the anti-contractile capacity of aPVAT in this high-output state. Through this functional switch, aPVAT sympathetic nerves therefore play a critical amplifying role in the systemic sympathetic vicious cycle. This functional significance aligns with the growing recognition of aortic BP as a critical predictor of hypertensive disease and reveals a previously unrecognized complexity in sympathetic vascular regulation.

### Therapeutic implications of aPVAT-specific neuromodulation

The management of hypertension driven by SNS hyperactivation remains a therapeutic challenge. Two key interventions, renal denervation (RDN) and SG blockade (SGB), both target sympathetic overactivity. RDN involves catheter-based ablation of renal nerves to reduce renin-angiotensin system activity [46], thereby lowering BP. Similarly, SGB inhibits sympathetic output to the heart and upper body via pharmacological blockade of the SG, an approach clinically used for refractory hypertension [47]. Notably, our data provide a foundation for potential functional implications: while the mechanism of SGB has traditionally been attributed to cardiac sympathetic suppression [48], our findings indicate that it may also partially block sympathetic output to aPVAT. This insight offers a novel explanation for anti-hypertensive effects of SGB, positioning aPVAT as a critical neuro−vascular interface in BP regulation. However, the application of RDN and SGB faces challenges including variable patient responsiveness and procedural risks [49, 50], which may due to a lack of cellular precision as they target entire ganglia containing mixed neuronal populations. A groundbreaking study recently revealed that the sympathetic system is not a monolithic entity but exhibits significant cellular and molecular diversity [51]. By focusing interventions on this aPVAT-localized innervation, it may be feasible to disrupt the pathological sympathetic drive to the vasculature while minimizing off-target effects on other organ systems. Furthermore, the functional implications of this localized sympathetic regulation may extend beyond the cold-exposure model to other conditions involving sympathetic overactivity, such as metabolic syndrome and chronic inflammation.

### Limitations of the study

Although we establish a potential link between sympathetic activation in aPVAT and the pressor response, several aspects require further investigation. First, since aPVAT is located in a core temperature region, how aPVAT senses and responds to the cold stimulus remains an intriguing area for future study. Second, the relationship between dynamic NE storage within PVAT and the activation of sympathetic nerves is not fully elucidated. Third, from a methodological perspective, directly targeting the innervation of the aortic arch adipose tissue may provide more precise causal evidence and could strengthen the translational foundation.

## Materials and methods

### Animals

All experimental procedures and surgical procedures in mice were performed in compliance with the protocols approved by the Institutional Animal Care and Use Committee of Shanghai Medical College, Fudan University. Male C57BL/6 mice (aged 6–8 weeks) were purchased from GemPharmatech. They were maintained on the 12-h light/12-h dark cycle, with chow diet and water available.

### Antibodies and dyes

Primary antibodies and dyes used in this study included anti-sheep-TH (1:200 dilution, Sigma, ab1542), anti-rabbit-perilipin (1:200 dilution, Santa Cruz Biotechnology, sc-67164), anti-rabbit-synaptophysin (1:200 dilution, Gene Tex, gtx100865), anti-rabbit-TH (1:1000 dilution, Abclonal, A5079), anti-rabbit-UCP1 (1:1000 dilution, Abcam, ab155117), anti-rabbit-PGC1α (1:1000 dilution, Abcam, ab54481), and anti-mouse-β-actin (1:1000 dilution, Santa Cruz Biotechnology, sc-47778). Secondary antibodies included donkey anti-rabbit IgG Alexa Fluor 594 (1:200 dilution, Yeasen, 34212ES60) and donkey anti-sheep IgG Alexa Fluor 647 (1:200 dilution, Yeasen, 34913ES).

### Whole-mount immunostaining and tissue clearing

The procedure was based on the published iDISCO+ technique [52]. Mice under indicated experimental conditions were anesthetized and perfused with ice-cold phosphate-buffered saline (PBS). The aPVAT was then micro-dissected and fixed in 1% paraformaldehyde (PFA)/10% sucrose/PBS at 4°C overnight. Following fixation, tissues were washed with PBS for 1 h three times. The tissues were then progressively dehydrated at room temperature in 20%, 40%, 60%, and 80% methanol (diluted in ddH_2_O) for 30 min each, and 100% methanol for 30 min twice. Subsequently, tissues were bleached in 5% H_2_O_2_ (1 volume of H_2_O_2_/4 volumes of methanol) at 4°C for 24 h and rehydrated at room temperature through reverse methanol gradients (80%, 60%, 40%, and 20% methanol diluted in ddH_2_O for 30 min each). Permeabilization was achieved by incubating tissues in PBS/0.2% Triton X-100/20% DMSO/0.3 mol/L glycine at 37°C for 24 h. Nonspecific binding sites were blocked with PBS/0.2% Triton X-100/10% DMSO/5% donkey serum at 37°C for 24 h. Primary antibodies (diluted 1:200 in PBS/0.2% Tween-20/5% DMSO/5% donkey serum) were applied at 37°C for 72 h, and washed in PBS/0.2% Tween-20 at 37°C for 1 h five times. Alexa Fluor-conjugated secondary antibodies (1:200 dilution in PBS/0.2% Tween-20/5% donkey serum) were then incubated at 37°C for 48 h, followed by five 2-h washes in PBS/0.2% Tween-20. Immunolabeled PVAT samples were embedded in 1% agarose blocks prepared in PBS. The tissue blocks were dehydrated in tubes at room temperature through a methanol series (20%, 40%, 60%, 80%, and 100% methanol diluted in ddH_2_O for 1 h each). Samples were then treated with the mixture of dichloromethane (Sigma 270997)/methanol (2 volumes/1 volume) for 3 h and 100% dichloromethane for 15 min twice. After this process, complete delipidation was achieved by settling the samples to the bottom of the tube. If necessary, the incubation in 100% dichloromethane was prolonged. Final clearing was performed twice in 100% dibenzyl ether (Sigma 108014) for 1 h to become ready for the volume fluorescence imaging. The fully cleared samples were stored in dibenzyl ether at room temperature, protected from light, and imaged as soon as possible to minimize fluorescence quenching.

### Volume imaging and image processing

Optically cleared aPVAT was imaged on the light sheet microscope (LS-18, Nuohai Life Science, Shanghai, China). For imaging, the tissue blocks were immersed in the chamber filled with 100% dibenzyl ether. Imaging was performed at 1× effective magnification (6.3× zoom). The samples were scanned from the right side using three combined light sheets, with a step-size of 2 μm.

Imaris was used to reconstruct the image stacks obtained from the volume imaging. For visualization purposes in figures and movies, a gamma correction between 0.5 and 1.0 was applied to the raw image data. To quantify the sympathetic innervation, the Imaris Surface algorithm was used to reconstruct the aPVAT volume, and the Imaris Filament algorithm was used to reconstruct sympathetic nerve fibers. This semi-automated tracing allowed for the quantification of key morphological parameters, including fiber density (calculated as the volume of filaments per unit volume of the surface), length, and diameter. Movies of the image stacks were generated at a frame rate of 30 fps.

### Cold challenge

The mice were transferred from room temperature (22°C–23°C) to 4°C to be cold-challenged. (i) To determine the expression levels of TH, PVAT was acutely dissected out at 72 h after the cold challenge, and total RNA was extracted by TRIZOL (Life Technologies, Carlsbad, CA, USA) and processed for SYBR Green (Thermo Fisher Scientific) qPCR analysis. (ii) To determine the protein levels of TH, PVAT was acutely dissected out at 72 h after the cold challenge, and homogenized in urea/SDS buffer (50 mmol/L Tris-Cl at pH 6.8, 8 mol/L urea, 10% SDS, and 50 mmol/L DTT) for immunoblot ana­lysis as previously described [53]. (iii) To examine the appearance of the cold-induced adipocytes, mice were perfused with PBS followed by PBS/1% PFA. PVAT was post-fixed in PBS/1% PFA at 4°C overnight and processed for paraffin sectioning and H&E staining.

To assess the impact of cold exposure on BP dynamics, mice were transferred from room temperature (22°C–23°C) to 16°C or 4°C to be cold-challenged for 4 h (10:00 a.m. to 2:00 p.m.) daily over 2 weeks. On the day of BP measurement, the mice were subjected to cold exposure from 3:00 p.m. to 7:00 p.m. BP and HR were measured via tail-cuff system (CODA, Kent Scientific, USA).

### Surgical procedures

For the retrograde tracing of ganglia, the wild-type mice were anesthetized and a dorsal midline incision in the skin was made. The skin was then separated to the sides to expose the spine. Alexa594-conjugated CTB (Life Technologies) was injected (5 μg/μL in saline, 1 μL per injection) to four different positions of each fat pad along the spine at a 30° low angle, after which the skin incision was sutured. Seven days after the CTB injection, the ganglia were harvested as reported [54, 55] from the mice and processed for cryosectioning and immunostaining. CTB-labeled neurons in the ganglia were visualized by fluorescence microscopy.

To pharmacologically ablate the sympathetic arborizations within PVAT, 10 μL 6-OHDA (MCE, 10 μg/μL freshly prepared in PBS) was evenly distributed along aPVAT using procedure analogous to the tracing injections, after which the skin incision was sutured. Six days after the pharmacologic ablation, the mice were utilized for further experiments.

### Cold-induced hypertension model

Male C57BL/6 mice (6–8 weeks old) were randomly allocated into one of the following experimental groups: (i) control group; (ii) cold-exposure group; (iii) 6-OHDA-abalation and cold-exposure group. To establish the cold-induced hypertension model, mice were exposed to 4°C for 4 h per day over a period of two weeks, while control mice were maintained at room temperature (22°C–23°C). The development of hypertension was confirmed by a significant increase in SBP and DBP.

### Quantitative PCR

Total RNA was extracted from aPVAT samples using TRIZOL (Life Technologies, Carlsbad, CA, USA) according to the manufacturer protocol. RNA concentrations and purity were determined using a microvolume spectrophotometer (Berthold, Germany). For cDNA synthesis, 2 µg total RNA was reverse transcribed using the RevertAid First Strand cDNA Synthesis Kit (Thermo Fisher Scientific, USA). Then, the levels of indicated mRNAs were analyzed using SYBR Green Master Mix on the QuantStudio real-time PCR system. The relative expression of target genes was calculated using the 2^−△△Ct^ method, with 18S mRNA for normalization. Data were expressed as fold change relative to the control group.

### Histological analysis

For morphological assessment, aPVAT samples were fixed in 4% PFA, embedded in paraffin, and sectioned at 5-µm thickness. For H&E staining, sections were deparaffinized in xylene, rehydrated through a graded ethanol series, and stained with hematoxylin for 5 min. After differentiation and bluing, sections were counterstained with eosin for 3 min, followed by dehydration, clearing, and mounting with neutral balsam. Stained sections were exa­mined under light microscope to evaluate general tissue morphology and adipocyte size.

For immunohistochemical (IHC) analysis, after deparaffinization, rehydration, and antigen retrieval in 0.01 mol/L citrate buffer (pH 6.0) using microwave, endogenous peroxidase activity was quenched with 3% H_2_O_2_. Sections were blocked with goat serum and then incubated with primary antibodies against UCP1 (Abcam, ab155117, 1:100) or TH (Abclonal, A5079, 1:100) overnight at 4°C. Binding was detected using a rabbit ABC Staining System (Vector Laboratories) according to the manufacturer’s instructions, with 3,3'-diaminobenzidine (DAB) as the chromogen. Sections were counterstained with hematoxylin, dehydrated, cleared, and mounted.

For IF analysis, a similar antigen retrieval and blocking procedure was followed. Sections were incubated with the same primary antibodies overnight at 4°C, followed by appropriate Alexa Fluor-conjugated secondary antibodies. Nuclei were counterstained with 4'-6-diamidino-2-phenylindole (DAPI). Images were captured using a fluorescence microscope.

### BP measurement

BP and HR were measured by a noninvasive tail-cuff system (CODA, Kent Scientific, USA). Mice were acclimated to the restraint device for 7 days, followed by 2 days of measurement training before formal experiments. Measurements were conducted during the daytime (10:00 a.m. to 2:00 p.m.), with 30 consecutive cycles recorded after 15-min stabilization period. Data were collected on two consecutive days at each time point, and the more stable results of the second day were used for analysis. All mice were measured at 1, 0, 6, 7, 13, and 14 days after cold exposure.

### Quantification and statistical analysis

All data are presented as the mean ± SEM. GraphPad Prism 8.0 (Graph-Pad Software, San Diego, CA, USA) was used for data visualization. Statistical comparisons between two groups were performed by unpaired Student’s *t*-tests, and comparisons among three or more groups were performed by ANOVA. For experiments with a small sample size (*n *= 3), nonparametric tests were employed. In all statistical comparisons, a *P* value of < 0.05 was considered as a statistically significant difference. The numbers per group in the figure legends refer to the number of mice per group.

## Supplementary Material

loag015_Supplementary_Data

## Data Availability

The data that support the findings of this study are available from the corresponding author upon reasonable request.

## References

[1] Qi XY , Qu SL, Xiong WH et al Perivascular adipose tissue (PVAT) in atherosclerosis: a double-edged sword. Cardiovasc Diabetol 2018;17:134. 30305178 10.1186/s12933-018-0777-x PMC6180425

[2] Cheng CK , Bakar HA, Gollasch M et al Perivascular adipose tissue: the sixth man of the cardiovascular system. Cardiovasc Drugs Ther 2018;32:481–502. 30171461 10.1007/s10557-018-6820-z PMC7101924

[3] Sena CM , Pereira A, Fernandes R et al Adiponectin improves endothelial function in mesenteric arteries of rats fed a high-fat diet: role of perivascular adipose tissue. Br J Pharmacol 2017;174:3514–26. 28236429 10.1111/bph.13756 PMC5610162

[4] Xia N , Horke S, Habermeier A et al Uncoupling of endothelial nitric oxide synthase in perivascular adipose tissue of diet-induced obese mice. Arterioscler Thromb Vasc Biol 2016;36:78–85. 26586660 10.1161/ATVBAHA.115.306263

[5] Nosalski R , Guzik TJ. Perivascular adipose tissue inflammation in vascular disease. Br J Pharmacol 2017;174:3496–513. 28063251 10.1111/bph.13705 PMC5610164

[6] Xia N , Li H. The role of perivascular adipose tissue in obesity-induced vascular dysfunction. Br J Pharmacol 2017;174:3425–42. 27761903 10.1111/bph.13650 PMC5610151

[7] Mu WJ , Song YJ, Yang LJ et al Bone morphogenetic protein 4 in perivascular adipose tissue ameliorates hypertension through regulation of angiotensinogen. Front Cardiovasc Med 2022;9:1038176. 36457800 10.3389/fcvm.2022.1038176 PMC9707298

[8] Koenen M , Becher T, Pagano G et al Ablation of *Prdm16* and beige fat identity causes vascular remodeling and elevated blood pressure. Science 2026;391:306–13. 41538429 10.1126/science.ady8644 PMC13229588

[9] Ahmad MF , Ferland D, Ayala-Lopez N et al Perivascular adipocytes store norepinephrine by vesicular transport. Arterioscler Thromb Vasc Biol 2019;39:188–99. 30567483 10.1161/ATVBAHA.118.311720 PMC6344267

[10] Ayala-Lopez N , Martini M, Jackson WF et al Perivascular adipose tissue contains functional catecholamines. Pharmacol Res Perspect 2014;2:e00041. 24904751 10.1002/prp2.41 PMC4041285

[11] Greaney JL , Kenney WL, Alexander LM. Neurovascular mechanisms underlying augmented cold-induced reflex cutaneous vasoconstriction in human hypertension. J Physiol 2017;595:1687–98. 27891612 10.1113/JP273487 PMC5330863

[12] Cuspidi C , Ochoa JE, Parati G. Seasonal variations in blood pressure: a complex phenomenon. J Hypertens 2012;30:1315–20. 22706390 10.1097/HJH.0b013e328355d7f9

[13] Chistiakov DA , Ashwell KW, Orekhov AN et al Innervation of the arterial wall and its modification in atherosclerosis. Auton Neurosci 2015;193:7–11. 26164815 10.1016/j.autneu.2015.06.005

[14] Parati G , Esler M. The human sympathetic nervous system: its relevance in hypertension and heart failure. Eur Heart J 2012;33:1058–66. 22507981 10.1093/eurheartj/ehs041

[15] Mohanta SK , Peng L, Li Y et al Neuroimmune cardiovascular interfaces control atherosclerosis. Nature 2022;605:152–9. 35477759 10.1038/s41586-022-04673-6

[16] Ayala-Lopez N , Martini M, Jackson WF et al Perivascular adipose tissue contains functional catecholamines. Pharmacol Res Perspect 2014;2:e00041. 24904751 10.1002/prp2.41 PMC4041285

[17] Pizzinat N , Marti L, Remaury A et al High expression of monoamine oxidases in human white adipose tissue: evidence for their involvement in noradrenaline clearance. Biochem Pharmacol 1999;58:1735–42. 10571247 10.1016/s0006-2952(99)00270-1

[18] Ayala-Lopez N , Jackson WF, Burnett R et al Organic cation transporter 3 contributes to norepinephrine uptake into perivascular adipose tissue. Am J Physiol Heart Circ Physiol 2015;309:H1904–14. 26432838 10.1152/ajpheart.00308.2015 PMC4698381

[19] Saxton SN , Ryding KE, Aldous RG et al Role of sympathetic nerves and adipocyte catecholamine uptake in the vasorelaxant function of perivascular adipose tissue. Arterioscler Thromb Vasc Biol 2018;38:880–91. 29496660 10.1161/ATVBAHA.118.310777

[20] Hanscom M , Morales-Soto W, Watts SW et al Innervation of adipocytes is limited in mouse perivascular adipose tissue. Am J Physiol Heart Circ Physiol 2024;327:H155–81. 38787382 10.1152/ajpheart.00041.2024 PMC11380956

[21] Huesing C , Zhang R, Gummadi S et al Organization of sympathetic innervation of interscapular brown adipose tissue in the mouse. J Comp Neurol 2022;530:1363–78. 34837221 10.1002/cne.25281 PMC9010363

[22] Lyons CE , Razzoli M, Larson E et al Optogenetic-induced sympathetic neuromodulation of brown adipose tissue thermogenesis. FASEB J 2020;34:2765–73. 31908033 10.1096/fj.201901361RR PMC7306786

[23] Jiang H , Ding X, Cao Y et al Dense intra-adipose sympathetic arborizations are essential for cold-induced beiging of mouse white adipose tissue. Cell Metab 2017;26:686–92.e3. 28918935 10.1016/j.cmet.2017.08.016

[24] Cao Q , Wang S, Wang H et al Fatty acids rescue the thermogenic function of sympathetically denervated brown fat. Biomolecules 2021;11:1428. 34680061 10.3390/biom11101428 PMC8533276

[25] Zeng W , Pirzgalska RM, Pereira MMA et al Sympathetic neuro-adipose connections mediate leptin-driven lipolysis. Cell 2015;163:84–94. 26406372 10.1016/j.cell.2015.08.055 PMC7617198

[26] Wehrwein EA , Orer HS, Barman SM. Overview of the anatomy, physiology, and pharmacology of the autonomic nervous system. Compr Physiol 2016;6:1239–78. 27347892 10.1002/cphy.c150037

[27] Scott-Solomon E , Boehm E, Kuruvilla R. The sympathetic nervous system in development and disease. Nat Rev Neurosci 2021;22:685–702. 34599308 10.1038/s41583-021-00523-y PMC8530968

[28] Chi J , Crane A, Wu Z et al Adipo-Clear: a tissue clearing method for three-dimensional imaging of adipose tissue. J Vis Exp 2018;137:58271. 10.3791/58271 PMC6126572 30102289

[29] Reutersberg B , Pelisek J, Ouda A et al Baroreceptors in the aortic arch and their potential role in aortic dissection and aneurysms. JCM 2022;11:1161. 35268252 10.3390/jcm11051161 PMC8911340

[30] Sun H , Li DP, Chen SR et al Sensing of blood pressure increase by transient receptor potential vanilloid 1 receptors on baroreceptors. J Pharmacol Exp Ther 2009;331:851–9. 19726694 10.1124/jpet.109.160473 PMC2784714

[31] Abu Bakar H , Robert Dunn W, Daly C et al Sensory innervation of perivascular adipose tissue: a crucial role in artery vasodilatation and leptin release. Cardiovasc Res 2017;113:962–72. 28371926 10.1093/cvr/cvx062

[32] Chang HH , Yang SSD, Chang SJ. Perivascular adipose tissue modulation of neurogenic vasorelaxation of rat mesenteric arteries. J Cardiovasc Pharmacol 2020;75:21–30. 31633584 10.1097/FJC.0000000000000761

[33] Alahmad B , Khraishah H, Royé D et al Associations between extreme temperatures and cardiovascular cause-specific mortality: results from 27 countries. Circulation 2023;147:35–46. 36503273 10.1161/CIRCULATIONAHA.122.061832 PMC9794133

[34] Chen Z , Liu P, Xia X et al The underlying mechanisms of cold exposure-induced ischemic stroke. Sci Total Environ 2022;834:155514. 35472344 10.1016/j.scitotenv.2022.155514

[35] McEniery CM , Cockcroft JR, Roman MJ et al Central blood pressure: current evidence and clinical importance. Eur Heart J 2014;35:1719–25. 24459197 10.1093/eurheartj/eht565 PMC4155427

[36] Kario K , Kanegae H, Oikawa T et al Hypertension is predicted by both large and small artery disease. Hypertension 2019;73:75–83. 30571549 10.1161/HYPERTENSIONAHA.118.11800

[37] Bulloch JM , Daly CJ. Autonomic nerves and perivascular fat: interactive mechanisms. Pharmacol Ther 2014;143:61–73. 24560685 10.1016/j.pharmthera.2014.02.005

[38] Quarti-Trevano F , Seravalle G, Grassi G. Clinical relevance of the sympathetic-vascular interactions in health and disease. Biomedicines 2021;9:1007. 34440211 10.3390/biomedicines9081007 PMC8394495

[39] Edwards DG , Gauthier AL, Hayman MA et al Acute effects of cold exposure on central aortic wave reflection. J Appl Physiol (1985) 2006;100:1210–4. 16223975 10.1152/japplphysiol.01154.2005

[40] Gentilin A , Rakobowchuk M, Mourot L. Sex-specific responses of central artery stiffness to cold pressor test-mediated sympathetic activation. Physiol Behav 2025;289:114755. 39577791 10.1016/j.physbeh.2024.114755

[41] Bruno RM , Ghiadoni L, Seravalle G et al Sympathetic regulation of vascular function in health and disease. Front Physiol 2012;3:284. 22934037 10.3389/fphys.2012.00284 PMC3429057

[42] Charkoudian N , Wallin BG. Sympathetic neural activity to the cardiovascular system: integrator of systemic physiology and interindividual characteristics. Compr Physiol 2014;4:827–50. 10.1002/cphy.c130038 24715570

[43] Löhn M , Dubrovska G, Lauterbach B et al Periadventitial fat releases a vascular relaxing factor. FASEB J 2002;16:1057–63. 12087067 10.1096/fj.02-0024com

[44] Bussey CE , Withers SB, Saxton SN et al β_3_-adrenoceptor stimulation of perivascular adipocytes leads to increased fat cell-derived NO and vascular relaxation in small arteries. Br J Pharmacol 2018;175:3685–98. 29980164 10.1111/bph.14433 PMC6109217

[45] Chang L , Villacorta L, Li R et al Loss of perivascular adipose tissue on peroxisome proliferator–activated receptor-γ deletion in smooth muscle cells impairs intravascular thermoregulation and enhances atherosclerosis. Circulation 2012;126:1067–78. 22855570 10.1161/CIRCULATIONAHA.112.104489 PMC3493564

[46] Kario K. Catheter-based renal denervation ready for the management of hypertension: evidence, challenges, and perspectives. J Am Heart Assoc 2024;13:e037099. 39140342 10.1161/JAHA.124.037099 PMC11963923

[47] Ganesh A , Qadri YJ, Boortz-Marx RL et al Stellate ganglion blockade: an intervention for the management of ventricular arrhythmias. Curr Hypertens Rep 2020;22:100. 33097982 10.1007/s11906-020-01111-8 PMC7646199

[48] Cha Y , Li X, Yang M et al Stellate ganglion block and cardiac sympathetic denervation in patients with inappropriate sinus tachycardia. J Cardiovasc Electrophysiol 2019;30:2920–8. 31625219 10.1111/jce.14233 PMC6973270

[49] Denker MG , Cohen DL. Resistant hypertension and renal nerve denervation. Methodist Debakey Cardiovasc J 2015;11:240–4. 27057294 10.14797/mdcj-11-4-240 PMC4814011

[50] Wen S , Chen L, Wang TH et al The efficacy of ultrasound-guided stellate ganglion block in alleviating postoperative pain and ventricular arrhythmias and its application prospects. Neurol Sci 2021;42:3121–33. 34008041 10.1007/s10072-021-05300-4

[51] Wang T , Teng B, Yao DR et al Organ-specific sympathetic innervation defines visceral functions. Nature 2025;637:895–902. 39604732 10.1038/s41586-024-08269-0

[52] Renier N , Wu Z, Simon DJ et al iDISCO: a simple, rapid method to immunolabel large tissue samples for volume imaging. Cell 2014;159:896–910. 25417164 10.1016/j.cell.2014.10.010

[53] Mu W , Qian S, Song Y et al BMP4-mediated browning of perivascular adipose tissue governs an anti-inflammatory program and prevents atherosclerosis. Redox Biol 2021;43:101979. 33895484 10.1016/j.redox.2021.101979 PMC8099561

[54] Sleigh JN , Weir GA, Schiavo GA. Simple, step-by-step dissection protocol for the rapid isolation of mouse dorsal root ganglia. BMC Res Notes 2016;9:82. 26864470 10.1186/s13104-016-1915-8 PMC4750296

[55] Scherschel K , Bräuninger H, Glufke K et al Location, dissection, and analysis of the murine stellate ganglion. J Vis Exp 2020;166:e62026. 10.3791/62026 33427236

