# Supplementary material for: Three-dimensional mapping and functional analysis of sympathetic innervation in aortic perivascular adipose tissue
Source: Life Metab. 2026 Jun 8;5(4):loag015. doi: 10.1093/lifemeta/loag015 (PMC13342707; doi:10.1093/lifemeta/loag015)
Supplement: loag015_Supplementary_Data [file loag015_supplementary_data.zip › Supplementary Information - tu-clear.docx]

**Supplementary material**

**Three-dimensional mapping and functional analysis of sympathetic innervation in aortic perivascular adipose tissue**

Zhao-Ning Wang^1^, Yan-Jue Song^1^, Liang Tan^2^, Zhen-Yu Xu^1^, Ting Meng^1^, Dai-Chen Yao^1^, Yang Liu^1^, Shu-Wen Qian^1^, Qi-Qun Tang^1,*^, Yan Tang^1,†,*^

^1^Key Laboratory of Metabolism and Molecular Medicine, Ministry of Education, Department of Biochemistry and Molecular Biology of School of Basic Medical Sciences and Department of Endocrinology and Metabolism of Zhongshan Hospital, Fudan University, Shanghai 200032, China

^2^Department of Neurology, Jiaxing Hospital of Traditional Chinese Medicine Affiliated to Zhejiang Chinese Medical University, Jiaxing, Zhejiang 314001, China

^*^Corresponding authors. Key Laboratory of Metabolism and Molecular Medicine, Ministry of Education, Department of Biochemistry and Molecular Biology of School of Basic Medical Sciences and Department of Endocrinology and Metabolism of Zhongshan Hospital, Fudan University, Shanghai 200032, China. E-mail: [yantang@fudan.edu.cn](mailto:yantang@fudan.edu.cn) (Y.T.); [qqtang@shmu.edu.cn](mailto:qqtang@shmu.edu.cn) (Q.Q.T.)

^†^Lead contact

**
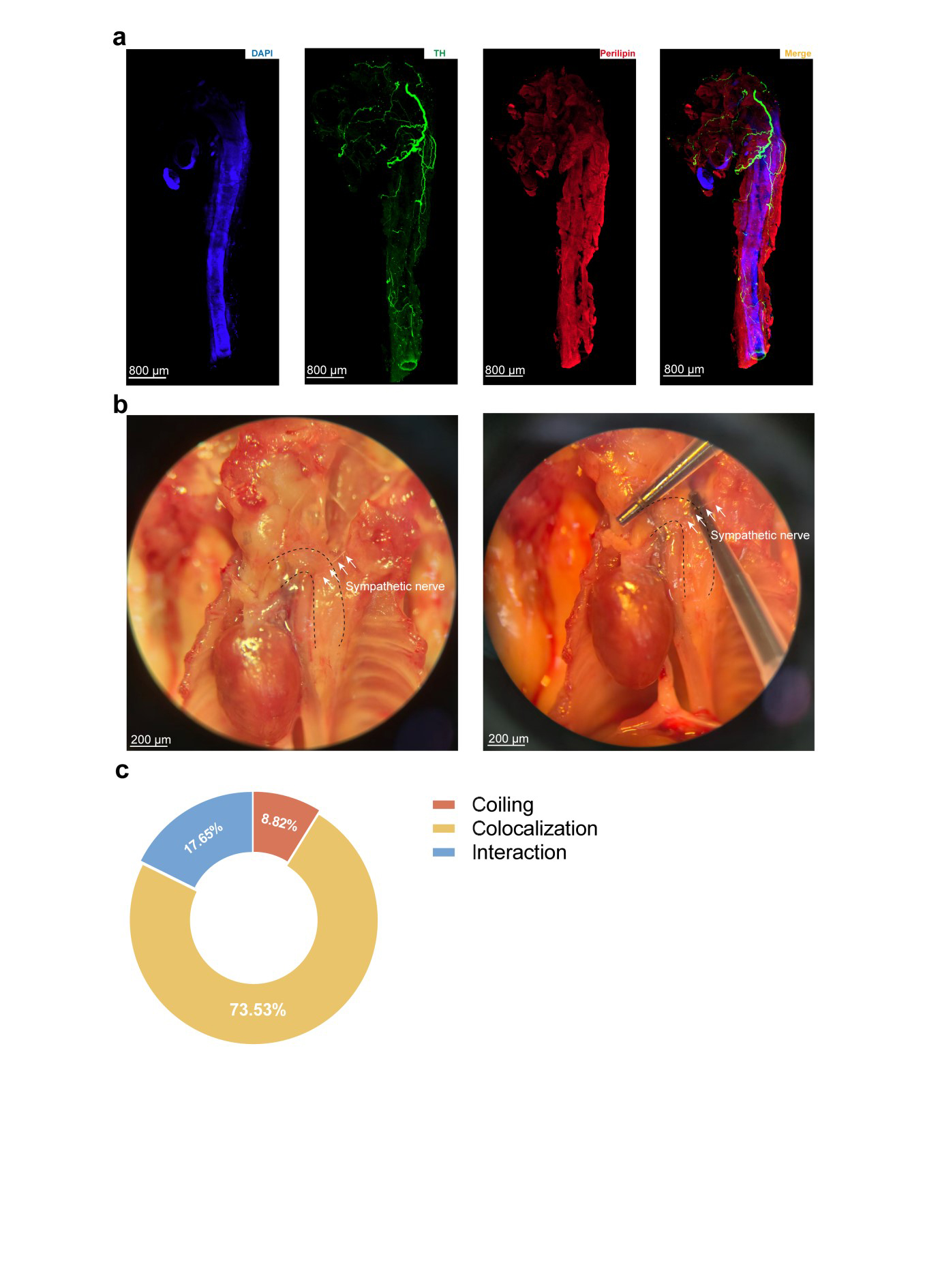
**

**Supplementary Figure S1** A hierarchically organized sympathetic network visualized by volume fluorescence imaging technique. (a) Volume image of aPVAT immunolabeled for DAPI (blue; blood vessel), tyrosine hydroxylase (TH, green; sympathetic nerves), and perilipin (red; adipocytes). (b) The primary sympathetic trunks observed under anatomical microscope. (c) Quantitative analysis on the three distribution patterns of sympathetic nerves in aPVAT.

**
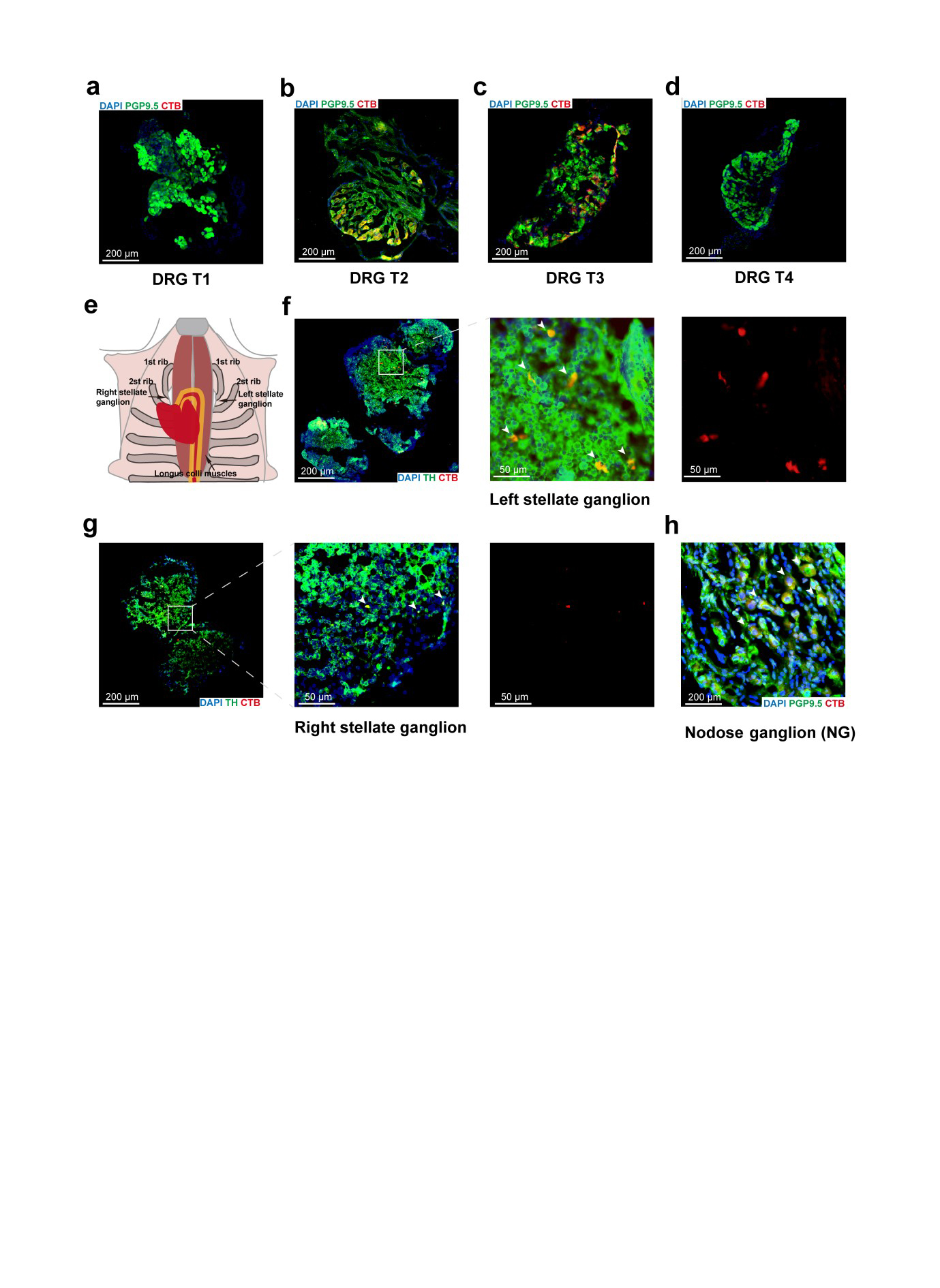
**

**Supplementary Figure S2** Retrograde tracing confirms dorsal root ganglia and stellate ganglion as origins of aPVAT innervation. (a−d) Representative image of CTB-labeled DRGs (red), colabeled with PGP9.5 (green) and DAPI (blue). (e−g) Representative image of a CTB-labeled (red) stellate ganglion section, colabeled with TH (green) and DAPI (blue). (h) Representative image of a CTB-labeled (red) nodose ganglion section, colabeled with PGP 9.5 (green) and DAPI (blue).


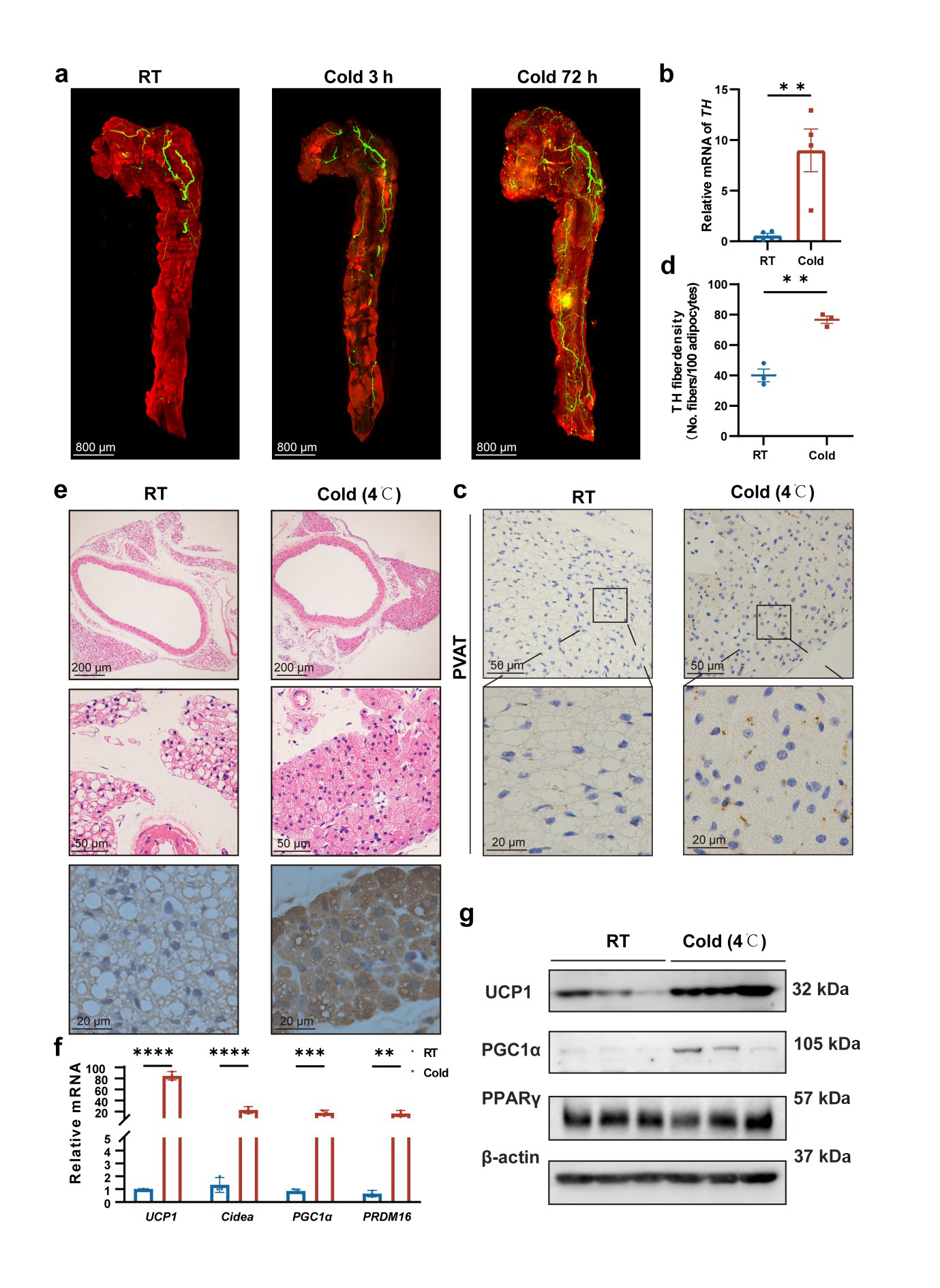


**Supplementary Figure S3** Cold exposure induces sympathetic activation and thermogenic response in aPVAT. (a) Representative 3D projections of aPVAT under room temperature (22℃−23℃) or cold exposure (4℃) for 3 h and 72 h, immunolabeled by anti-TH and anti-perilipin. (b) The qPCR analysis of TH levels in aPVAT from mice under room temperature (22℃−23℃) or cold exposure (4℃) for 72 h (*n* = 4). (c and d) Representative fluorography for H&E staining and quantification of TH fluorescent intensity in aPVAT. (e) Representative fluorography for H&E staining and quantification of UCP1 levels. (f) Relative mRNA levels of thermogenic genes (*UCP1*, *Cidea*, *PGC1α*, and *PRDM16*) in aPVAT of mice under room temperature (22℃−23℃) or cold exposure (4℃) for 72 h (*n* = 3). (g) Western blot analysis of thermogenic proteins (UCP1 and PGC1α) in aPVAT from mice under room temperature (22℃−23℃) or cold exposure (4℃) for 72 h.


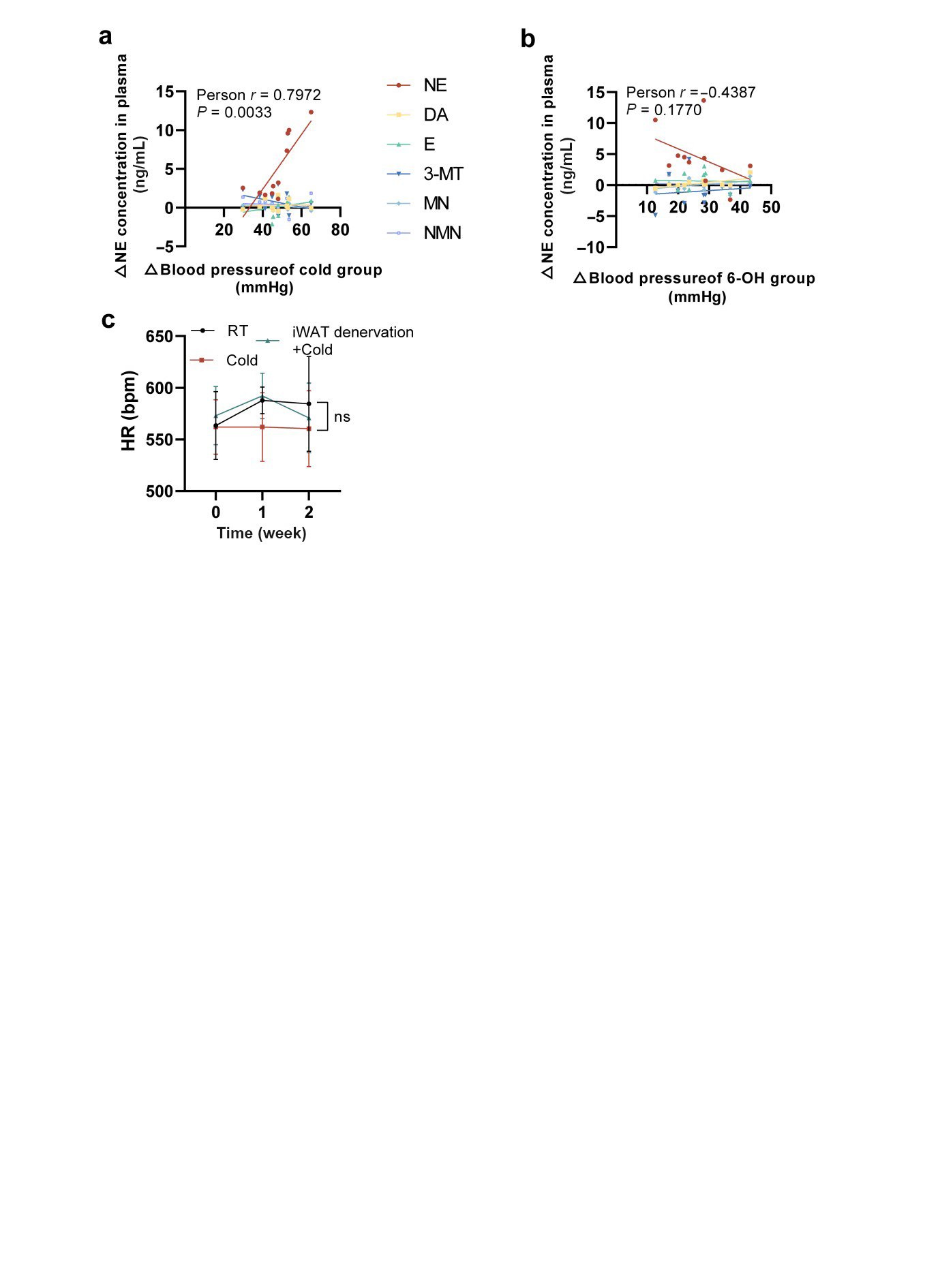


**Supplementary Figure S4** Blood pressure changes during cold exposure and correlation analysis between plasma catecholamine concentration and blood pressure. (a) Correlation analysis between blood pressure and plasma catecholamine concentration of cold exposure group: NE, DA, E, 3-MT, MN, and NMN (*n* = 11). (b) Correlation analysis between blood pressure and plasma catecholamine concentration of aPVAT denervation: NE, DA, E, 3-MT, MN, and NMN (*n* = 11). (C) Heart rate of iWAT denervation (*n* = 11).

**Supplementary Movie S1** Anti-tyrosine hydroxylase and perilipin whole-mount immunolabeling and volume fluorescence imaging of PVAT, related to Figure 1. PVAT of wild-type mice was processed for anti-tyrosine hydroxylase and perilipin immunolabeling and imaged on the light sheet microscope.
